# The corneal endothelium in an endotoxin-induced uveitis model: correlation between in vivo confocal microscopy and immunohistochemistry

**Published:** 2008-06-16

**Authors:** Liem Trinh, Françoise Brignole-Baudouin, Antoine Labbé, Mathilde Raphaël, Jean-Louis Bourges, Christophe Baudouin

**Affiliations:** 1 Department of Ophthalmology III, Quinze-Vingts National Ophthalmology Hospital, Paris, France; 2 INSERM UMR S 872, Cordeliers Biomedical Institute, Pierre et Marie Curie University – Paris 6, Paris Descartes University, Paris, France; 3 INSERM UMR S 592, Vision Institute, Pierre et Marie Curie University – Paris, France; 4 Department of Toxicology, Faculty of Biological and Pharmacological Sciences, University of Paris 5 René Descartes, Paris, France; 5 Department of Biostatistics and Medical Informatics, Saint-Louis Hospital, AP-HP, Paris, France

## Abstract

**Purpose:**

To analyze the involvement of the corneal endothelium in uveitis to better understand the formation mechanisms and the keratic precipitate composition. In vivo confocal microscopy images were correlated with ex vivo immunostaining of corneal endothelium from rat eyes with endotoxin-induced uveitis (EIU).

**Methods:**

EIU was induced in Lewis rats by lipopolysaccharide (LPS) injection. Slit-lamp examination and in vivo confocal microscopy were performed 6, 24, 48, 72, and 96 h after the LPS injection. One group of rats was killed at 24 h and the other rats at 96 h. Immunohistochemistry on corneal endothelium using antibodies to intercellular adhesion molecule (ICAM-1), phalloidin, CD68 (anti-macrophage), MCA967 (anti-granulocyte), T cell receptor alpha/beta (TCR alpha/beta; anti-lymphocyte), zonula occludens-1 (ZO-1), and occludin was performed on flat-mount corneas and was analyzed using a three dimensional (3D) laser confocal microscope.

**Results:**

In vivo confocal microscopy showed numerous hyperreflective round dots on the corneal endothelium, in the anterior chamber, and in the anterior stroma corresponding to inflammatory cells with a maximum at 24 h after the injection and detectable until the 96th hour. Upon immunostaining, corneal endothelial cells in rats with EIU overexpressed ICAM-1. ZO-1 and occludin had a lower endothelial expression and more heterogeneous distribution in EIU rats than in controls, showing disruption of endothelial cell junctions. Compared to controls, CD68, MCA967, and TCR alpha/beta expression was observed in corneas in rats with EIU. The two techniques showed a circular peripheral network of corneal vessels derived from a large circumferential vascular structure resembling the major arterial circle of iris where the inflammatory cells marginalized to infiltrate the anterior stroma.

**Conclusions:**

The correlation between in vivo confocal microscopy and ex vivo immunostaining helped to better understand in vivo confocal microscopy images. The two new techniques applied here were very effective and complementary in evaluating the corneal endothelium involvement in EIU. Based on these findings, in vivo confocal microscopy in clinical practice could be very helpful to better analyze keratic precipitates and corneal modifications in patients with uveitis.

## Introduction

Uveitis is an ocular pathology characterized by intraocular inflammation, which can be caused by several etiologies [1]. In clinical practice, the main issue is to evaluate severity of uveitis and determine its origin (infectious or inflammatory) to propose the most appropriate therapy. Involvement of the cornea, notably the endothelium, during uveitis has not been extensively studied even though it might participate in or constitute a target of ocular inflammation. Indeed, keratic precipitates are frequently observed in uveitis. They are small aggregates of inflammatory cells accumulated on the endothelial surface of the cornea [2]. Clinical aspects of these keratic precipitates can provide useful information on etiology and the degree of inflammatory activity. The precipitates are said to be granulomatous when they are large, and they might be secondary to etiologies such as sarcoidosis, tuberculosis, or toxoplasmosis. Other precipitates are called nongranulomatous when they are fine, for example in HLA-B27 associated anterior uveitis and Behçet’s syndrome, but are less helpful to formulate differential diagnosis than the granulomatous precipitates [3]. Little is known about the immune mechanisms of aggregation of these inflammatory cells because investigation of corneal endothelium in humans would be too aggressive and would require corneal samples. Recently, keratic precipitates were studied using in vivo confocal microscopy (CM), a noninvasive technique. Wertheim et al. [4] have also distinguished aspects of keratic precipitates of infectious causes from noninfectious etiologies. The aim of the present study was to investigate corneal endothelium alterations in an experimental model of uveitis (EIU) and describe the nature of inflammatory cells and mechanisms involved in the formation of keratic precipitates. We used original methods of investigations combining in vivo CM and ex vivo immunostaining of corneal endothelium on flat-mount corneas using a confocal microscope. Our laboratory has already used this in vivo CM on animals to compare the anatomy of animal corneas [5] and to assess ocular inflammation on a lipopolysaccharide-induced model of conjunctivitis in rabbits [6] and has combined the in vivo CM with immunohistological techniques to evaluate the proinflammatory and apoptotic effects of LPS in corneal injury models [7]. The endothelial modifications (expression of tight junction proteins, zonula occludens−1 [ZO-1] and occludin, overexpression of adhesion molecules such as intercellular adhesion molecule-1 [ICAM-1], and visualization of endothelial architecture with phalloidin staining of the cytoskeleton) and the nature of inflammatory cells involved (macrophages, neutrophils, and lymphocytes) were analyzed. This study could help to develop further analyses of keratic precipitates by in vivo CM and improve the understanding and interpretation of images observed in vivo in uveitis.

## Methods

### Animals

Eighteen inbred, male, adult, 8- to 10-week-old Lewis rats (Centre d’élevage R. Janvier, Le Genest-St-Isle, France) were used. Lipopolysaccharide (LPS) from *Escherichia coli* (Sigma Chemical, St Louis, MO) was dissolved in sterile pyrogen-free saline at 1 mg/ml. Uveitis was induced with a single subcutaneous injection of 150 μg LPS solution (1 mg/ml, 150 μl). Rats were divided into three groups of six rats: the first one was the control group where the rats were injected with sterile pyrogen-free saline at the same volume as the LPS injection, and the second and the third groups were injected with LPS. Rats in the first and second groups were sacrificed with an injection of pentobarbital at a lethal dose (sodium pentobarbital, Ceva Santé Animale, Libourne, France) at 24 h and the third one at 96 h. All animals were treated according to the Association for Research in Vision and Ophthalmology Resolution on the Human Use of Animals in Vision Research under the supervision of an independent health authority-accredited staff member for animal care and management.

### Slit-lamp examination and clinical score of endotoxin-induced uveitis

Slit-lamp examination was performed 6 and 24 h after the LPS injection. As previously described [8], the severity of uveitis was graded from 0 to 4 by a masked investigator as follows: 0=no inflammation; 1=discrete vasodilatation of the iris and the conjunctiva vessels; 2=moderate dilatation of the iris and the conjunctival vessels with moderate flare in the anterior chamber; 3=intense iridal hyperemia with intense flare in the anterior chamber; 4=the same clinical signs as 3 with fibrinous exudates in the pupillary area.

### In vivo confocal microscopy evaluation

The animals were examined with a laser scanning in vivo CM, the Heidelberg Retina Tomograph II Rostock Cornea Module (HRT II/RCM; Heidelberg Engineering®, Heidelberg, Germany), as described by Labbé et al. for small animals [5] and Liang et al. [6]. A diode laser with a wavelength of 670 nm is the laser source. The images have a resolution of 384×384 pixels, covering 400×400 μm with transversal and longitudinal optical resolution of 2 μm and 4 μm, respectively, and an acquisition time of 0.024 s. The depth of the optical section is manually controlled and allows exploration of all corneal layers. Under general anesthesia by an intraperitoneal injection of 2 mg/kg bodyweight of xylazine (Rompun 2%; Bayer Pharma, Puteaux, France) and 50 mg/kg bodyweight ketamine (Imalgène 500; Merial, Lyon, France) and after instillation of a drop of gel tear substitute (Lacrigel, carbomer 0.2%; Europhta, Monaco, Principauté de Monaco) on the tip of the objective lens, the rat eye was maintained in contact with the objective lens. Confocal microscopic images of each layer of cornea – superficial epithelium, basal epithelium, anterior and posterior stroma and endothelium – were recorded in each animal by focusing the microscope from the superficial epithelium to the endothelium on the central and peripheral cornea. The observation period for intravital microscopy was 96 h. In vivo CM was performed at 6, 24, 48h, 72, and 96 h after the injection of LPS for the uveitis groups and 6 and 24 h after the injection of sterile pyrogen-free saline for the control group. All images were analyzed and compared among groups. Endothelial density in cells/mm^2^ was quantified in the third group at the different time points (at 6, 24, 48, 72, and 96 h after the injection) and in the control group at 24 h using the program associated with the HRT II/RCM after acquisition and recording of endothelial images in the central cornea.

Data were expressed as the mean±standard deviation (SD). Outcomes were endothelial densities. Outcome variations versus time were analyzed using mixed models analysis of covariance (ANCOVA) with a fixed intervention effect, a fixed linear effect for time, and their interaction. Random subject effects were also added to the model. All tests were two-sided and at a 0.05 significance level. All analyses were performed using R 2.3.1 statistical software.

### Immunohistochemistry on flat-mount corneas and confocal microscopy

To characterize the inflammatory cells involved in this corneal infiltration and to study endothelial alterations occurring in this model, we correlated images of in vivo confocal microscopy with immunohistochemistry on flat-mount corneas analyzed with CM. As clinical scoring of endotoxin-induced uveitis (EIU) showed a maximal inflammatory level at 24 h [8], we chose this time point for immunohistochemical analysis. At the sacrifice times, 24 and 96 h after LPS injection, rat eyes were enucleated and the whole corneas were dissected, carved into four quarters, fixed in 4% paraformaldehyde for 2 h, and then rinsed with phosphate-buffered saline (PBS). Cellular membranes were permeabilized with 1% Triton X-100-PBS (Sigma Chemical, Saint Louis, MO) for 20 min and then washed with PBS. The quarters of cornea were incubated in microtubes at room temperature for 1 h with one of the following specific primary antibodies diluted in PBS containing 1% bovine serum albumin (BSA; Sigma Chemical, Saint Louis, MO):

-Monoclonal mouse anti-rat MCA967 staining granulocytes and erythrocytes (Serotec, Oxford, UK), diluted (1:20) in PBS containing 1% BSA.
-Anti-rat CD 68 MCA 341 R monoclonal mouse antibody, staining macrophages (Serotec) in a 1:100 dilution.
-Monoclonal mouse anti-rat TCR alpha/beta MCA 453 G, staining T lymphocytes (Serotec), diluted (1:25) in PBS containing 1% BSA.
-Monoclonal mouse anti-rat CD 54 ICAM-1 (Serotec), diluted (1:50) in PBS containing 1% BSA, staining intercellular adhesion molecule 1 (ICAM-1).
-Polyclonal rabbit anti-rat ZO-1 H300 (Santa Cruz Biotechnology, Santa Cruz, CA), staining tight junction protein Zonula Occludens-1 (ZO-1) in a 1:100 dilution.
-Anti-occludin H279 polyclonal rabbit antibody (Santa Cruz Biotechnology), staining tight junction protein occludin diluted (1:100) in PBS containing 1% BSA.
-Alexa Fluor 488 phalloidin (Invitrogen, Eugene, OR), a high-affinity probe for F-actin, staining cytoskeleton, diluted at 1:50.
-IgG1 mouse antibody for isotypic control (Serotec) in a 1:50 dilution.

After incubation with the specific primary antibodies, the cornea quarters were washed (twice for 5 min) with PBS and then incubated for 1 h in microtubes with secondary antibody, Alexa Fluor anti-mouse immunoglobulin G (Invitrogen,), diluted at 1:200 or with secondary antibody, Alexa Fluor anti-rabbit immunoglobulin G (Invitrogen), diluted at 1:100. The corneas were then rinsed in PBS (twice for 5 min), counterstained for 5 min with propidium iodide (Sigma Chemical,) in a 1:3000 dilution, and washed in PBS (once for 5 min). To localize the macrophages, granulocytes, and T lymphocytes in the cornea and to discriminate from the corneal endothelium, we proceeded to a double immunostaining with phalloidin and primary antibodies (CD 68, MCA967, TCR alpha/beta) revealed by a secondary antibody, Texas Red-associated anti-mouse immunoglobulin G (Jackson ImmunoResearch Laboratories, West Grove, PA), in a 1:100 dilution, and in this case, there was no propidium iodide adjunction. Finally, corneas were flat-mounted in PBS/glycerol (50/50), endothelial side on the top and epithelial side on the bottom. Corneas were examined using CM (Nikon PCM 200, Nikon France, Champigny sur Marne, France), and microphotographs were imaged in a 512×512-pixel size.

## Results

### Clinical scoring of endotoxin-induced uveitis

For the rats injected with LPS, the mean score for EIU was 2 at 6 h, 3 at 24 h, 2 at 48 h, 2 at 72 h, and 1 at 96 h. For control rats injected with sterile pyrogen-free saline, the mean score was 0 after 6 h and 24 h.

### In vivo confocal microscopy observation of the cornea in endotoxin-induced uveitis

**Figure 1:**
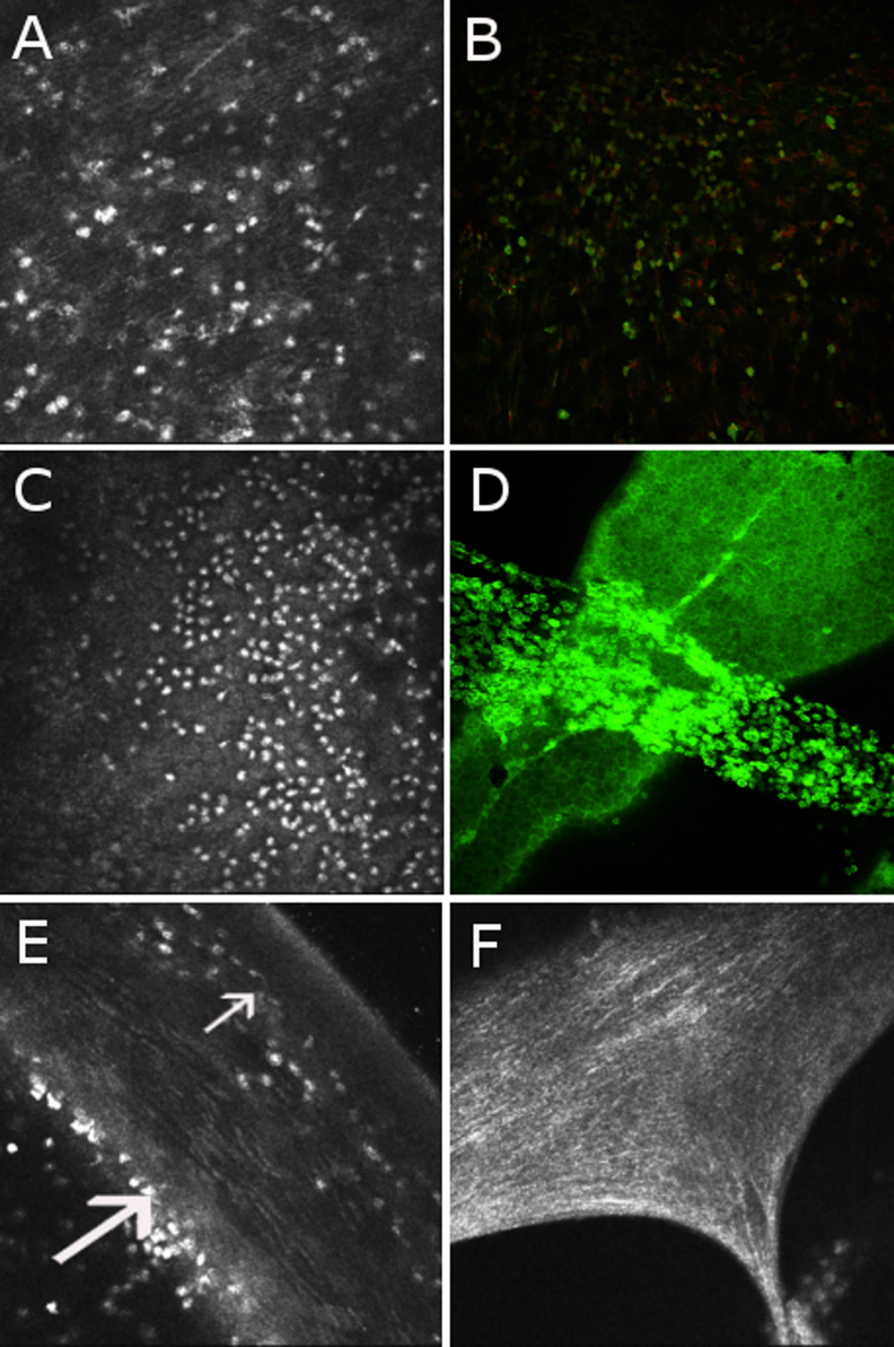
Correlation between in vivo confocal microscopy with immunohistochemistry on cornea in a model of Endotoxin Induced Uveitis. Heidelberg Retina Tomograph II (HRT II) in vivo confocal microscopy images of the cornea (400x400 μm) in a model of endotoxin-induced uveitis (**A**, **C**, **E**, and **F**) are correlated with ex vivo immunostaining images with Alexa Fluor 488 phalloidin staining cytoskeleton (in green) and counterstained with propidium iodide for nuclei (in red) on flat-mounted corneas that were analyzed by confocal microscopy of the same animals with a 20X enlargement (**B** and **D**). Inflammatory cells infiltrated the anterior stroma (**A** and **B**) and accumulated on the corneal endothelium, which appeared as a honeycomb-like mosaic (**C** and **D**). The anterior stroma (small arrow) and endothelium (large arrow) are shown on a corneal section by in vivo confocal microscopy (**E**). A clot of fibrin was individualized in the anterior chamber by HRT II (**F**).

**Figure 2:**
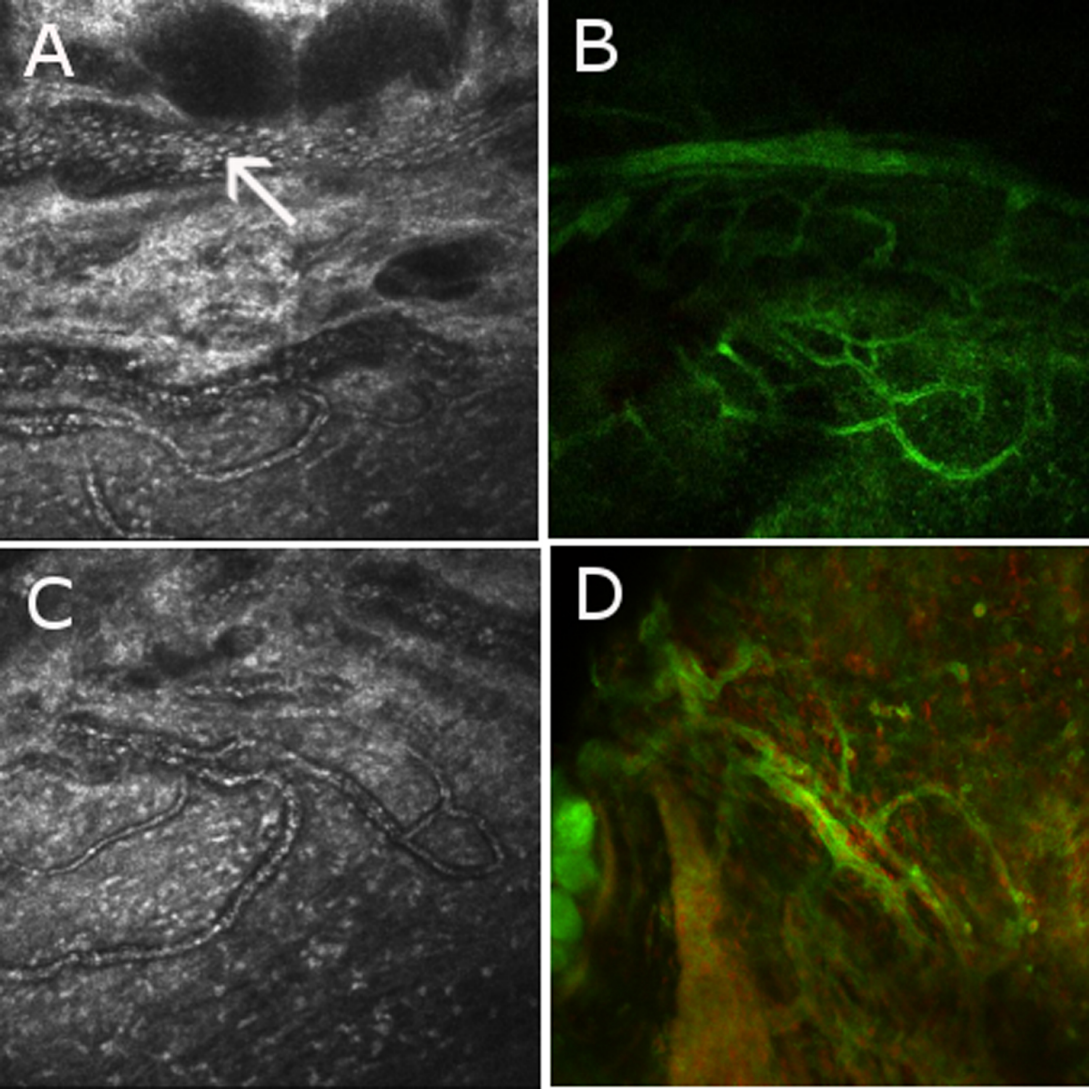
Correlation between in vivo confocal microscopy and immunohistochemistry on peripheral vascularisation in the corneal anterior stroma in a model of Endotoxin Induced Uveitis. **A**: In vivo confocal microscopic images (400 μm x 400 μm) of a circular peripheral network of vessels in the corneal anterior stroma, deriving from a large circumferential vascular structure resembling the major arterial circle of iris (arrow), are shown of rats injected with LPS. Margination and diapedesis of inflammatory cells can be visualized from these vessels toward the anterior stroma (**C**). These peripheral corneal vessels can also be shown in ex vivo immunostaining with phalloidin in the limbus of flat-mount corneas from rats injected with LPS (**B** and **D**).

The in vivo CM technique showed each layer of the cornea (superficial epithelium, basal epithelium, anterior stroma, posterior stroma, and endothelium) with normal aspects and no infiltration of inflammatory cells 6 and 24 h after injection of sterile pyrogen-free saline. Six hours after LPS injection, superficial and basal epithelium and posterior stroma presented no modifications, but infiltration of inflammatory cells, represented by hyperreflective round structures, had started to extend in the anterior stroma and along the endothelium. Inflammation peaked at 24 h after LPS injection as a major infiltration of inflammatory cells occurred in the anterior stroma, on the endothelium, and in the anterior chamber (Figure 1) and then progressively decreased after 24 h but persisted until 96 h after the LPS injection with a few inflammatory cells in the anterior stroma, on the endothelium, and in the anterior chamber. The endothelial distribution of inflammatory cells was diffuse on the whole surface of the cornea. Fibrin in the anterior chamber, adherent to the surface of the corneal endothelium, was shown with many inflammatory cells inside (Figure 1). A few hyperreflective round dots organized in clusters were also observed in the basal epithelium at 24 h. The posterior stroma was preserved from inflammatory infiltration, so we investigated the origin of inflammatory cells in the anterior stroma. Therefore, the limbus was particularly examined with CM, and numerous peripheral corneal loops of blood vessels constituting a circular network surrounding the corneal periphery with anastomosis were found in the limbus of rats injected with LPS as well as in the control rats (Figure 2). These vessels seemed to arise from a larger circumferential vascular structure resembling the major arterial circle of the iris. Rolling, defined by the slow down of cells which begins to roll along the inner surface of the vessels, and diapedesis of inflammatory cells could be visualized from these networks of vessels, which are attracted toward the anterior stroma, beginning at 6 h, maximal at 24 h, and decreasing until 96 h, but not in control eyes. The endothelial cell density progressively decreased after the LPS injection from the 6 h time point to the 72 h time point compared to the controls and then increased at 96 h, but there were no statistically significant differences (Table 1).

**Table 1:** Endothelial cell density (cells/mm^2^) quantified in control rats and in rats with EIU

	**Control**	**6 h**	**24 h**	**48 h**	**72 h**	**96 h**	p*
Endothelial cell density (cells/mm^2^)	3899.5 (72.6)	3552.3 (66.4)	3433 (85.6)	3134.3 (84.9)	3066.7 (79.8)	3409.2 (54.5)	0.6

The endothelial cell density, in cells/mm^2^ (mean±standard deviation), progressively decreased after the LPS injection from the 6th h to the 72nd h compared to the controls and then increased at 96 h, but there were no statistically significant differences. The asterisk indicates that the ANCOVA test was used for analysis.

### Immunohistochemical analysis and correlation with in vivo confocal microscopy

**Figure 3:**
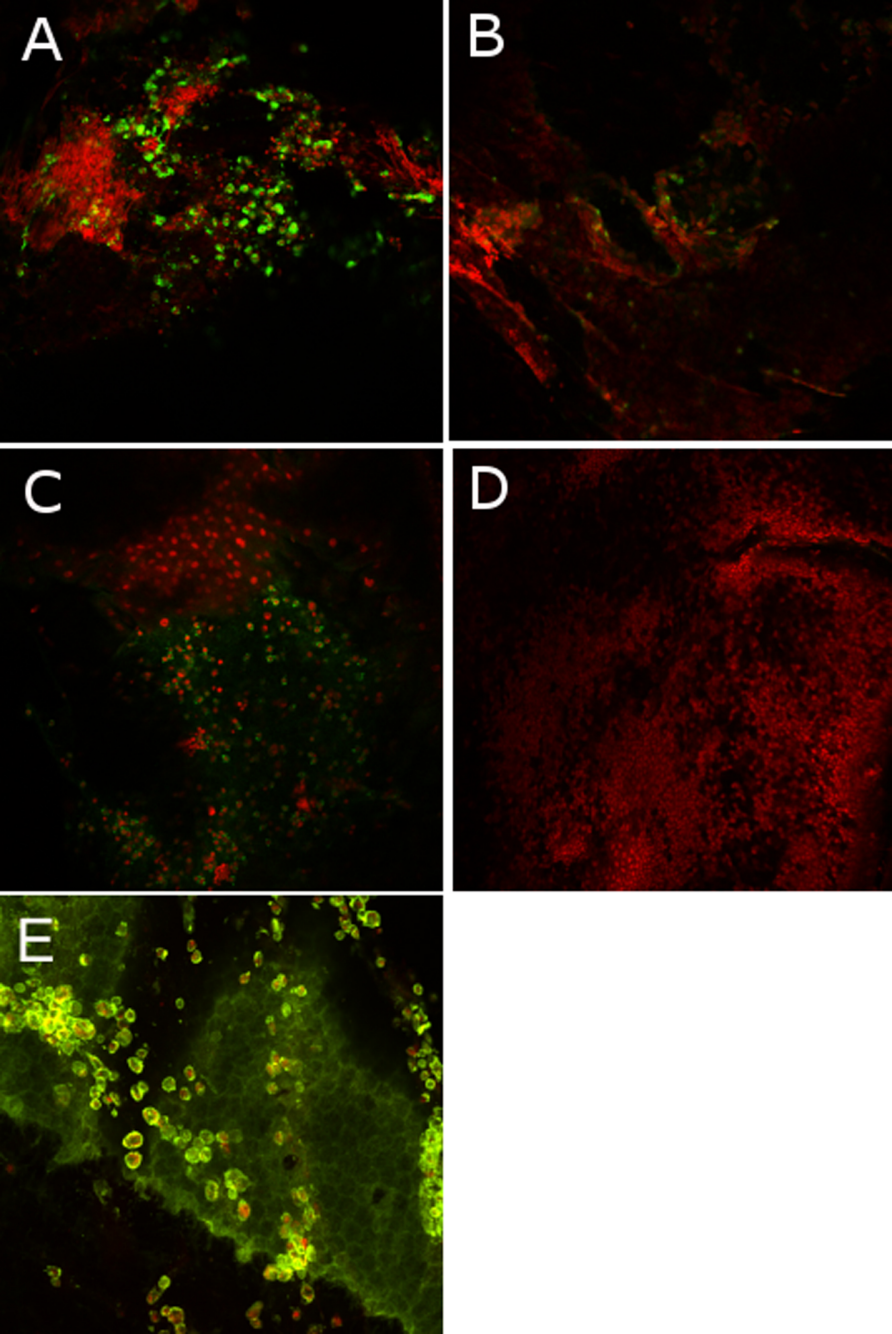
Immunofluorescence images on flat-mounted corneas in a model of endotoxin-induced uveitis analyzed with a 3D laser confocal microscope with a 20X enlargement. Immunostainings with anti-CD68 for macrophages (**A**), anti-TCR alpha/beta for lymphocytes (**B**), anti-MCA967 for granulocytes (**C**), and IgG1 mouse antibody for isotypic control (**D**) were revealed with secondary antibody, Alexa Fluor (in green) while the nuclear chromatin was stained with propidium iodide (in red). A double immunostaining with Alexa Fluor 488 phalloidin (in green), and antibody to CD68 (in red) was performed to localize the macrophages among the corneal layers, notably on the endothelium (**E**).

Immunohistochemistry on flat-mount corneas analyzed by CM confirmed results of in vivo CM and specified changes in the corneal endothelium in uveitis. Concerning cellular architecture, phalloidin staining showed the same images as the in vivo CM with the hexagonal structures of endothelial cells and cellular staining over its correspondence to inflammatory cells adhering to the endothelium (Figure 1). At 24 h, the inflammatory cells characterized by immunohistochemistry were macrophages, granulocytes, and T lymphocytes with specific primary antibodies, CD 68, MCA967, and TCR alpha/beta, respectively (Figure 3). At 24 h, mean cell counts could be quantified and 52% of macrophages (CD68 positive), 32% of granulocytes (MCA 967 positive) and 16% of T-lymphocytes (TCR alpha/beta positive) could be quantified among the total of immunostained cells in the same surface analyzed. Macrophages were localized in the different corneal layers, some in the epithelium but mostly in the anterior stroma and on the endothelium (Figure 3), and were correlated to in vivo CM images. Some granulocytes and a few T lymphocytes were distributed similarly in these layers in a smaller proportion. At 96 h, there were very few immunostained cells remaining at this time (data not shown), but macrophages still predominated.

**Figure 4:**
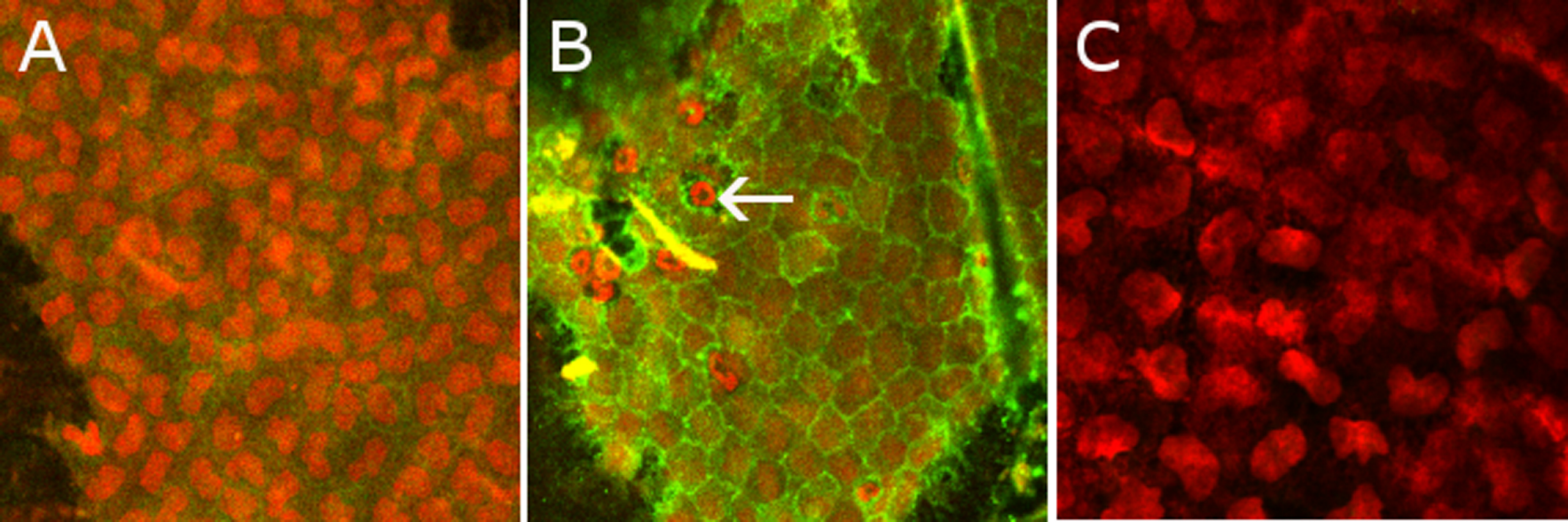
Double immunostaining with anti-CD54 ICAM-1 (in green) and with propidium iodide (in red) on corneas. The corneas were analyzed with a confocal microscope with a 200 X enlargement. Immunofluorescence of ICAM-1 is very weak in control animals (**A**) and was increased 24 h after the LPS injection (**B**). Some polymorphonuclear cells (arrow) are visible on the endothelium. Double immunofluorescence with IgG1 mouse antibody for isotypic control (revealed by secondary Alexa Fluor in green) and with propidium iodide (in red) was performed in EIU (**C**) with a 250X enlargement. Intensities of staining were the same for each assay.

Endothelial cellular modifications were analyzed regarding tight junction proteins and the adhesion molecule, ICAM-1. Fluorescence of ZO-1 and occludin was decreased in EIU compared to control rats in intercellular spaces (data not shown). On the contrary, ICAM-1 staining was clearly increased in EIU in comparison with control animals (Figure 4).

As peripheral corneal blood vessels could be described by in vivo CM to explain inflammatory cell infiltration in the anterior stroma, we particularly examined the corneolimbic junction in phalloidin immunostaining on flat-mount corneas and found networks of vessels similar to those observed using in vivo CM (Figure 2).

## Discussion

In uveitis, the role of the corneal endothelium has not been widely studied, and little is known about the mechanisms of keratic precipitate formulation, probably because the endothelium is hard to access, making investigation difficult. The original technique developed in this study to analyze the endothelium consisted in correlating ex vivo immunohistochemistry images on flat-mount corneas with in vivo confocal microscopy. Corneas were dissected, immunostained, reverted, and flat-mounted to have a full access to the endothelium so that different layers of the cornea could be studied by confocal microscopy. Comparing this technique with in vivo CM is quite useful because ex vivo immunostaining results improved interpretations of in vivo CM images.

Involvement of the corneal endothelium inside intraocular inflammation could therefore be defined. Endothelial modifications and mechanisms of formation and cellular composition of keratic precipitates were highlighted. Indeed, this study showed a break in the tight junction proteins, ZO-1 and occludin, in endothelial cells in EIU by immunofluorescence. Endothelial cellular density in uveitis progressively decreased after the 6 h time point until the 72 h time point (the minimal endothelial cellular density) and began to increase again at 96 h, but these differences were not statistically significant. With specular microscopy, some authors also demonstrated a decrease in cell density in uveitis compared with normal endothelium [9] also without a statistically significant difference [10]. Endothelial expression of adhesion molecules such as ICAM-1 has been previously displayed in experimental autoimmune uveitis (EAU) [11] and E-selectin in the EIU [12]. In the current article, we emphasized overexpression of ICAM-1 by endothelial cells and inflammatory cells in EIU, suggesting a probable active inflammatory cell aggregation mechanism against the endothelium to form keratic precipitates. The correlation of in vivo CM images showing hyperreflective round dots on endothelium and corresponding to inflammatory cells with immunohistochemistry on flat-mounted corneas helped to specify the exact cellular composition of keratic precipitates. Thus, macrophages, granulocytes, and T lymphocytes could be identified, and these results agreed with the nature of inflammatory cells infiltrating the anterior chamber in EIU in the literature [13]. Furthermore, granulocytes were described in the subendothelial plane of cornea in keratouveitis [14], and macrophages were found on the surface of the corneal endothelium in cytomegalovirus retinitis [15].

Endothelial changes represent only a part of the overall inflammatory corneal modifications occurring in uveitis that could be entirely analyzed with HRT II. Epithelial modifications and infiltration of inflammatory cells in the anterior stroma were imaged by in vivo CM, which confirmed the histological and electronic microscopic results of Behar-Cohen et al. [16] in EIU. Peripheral corneal blood vessels – probably coming from the major arterial circle of the iris – were observed by HRT II and in immunostaining to initiate margination of inflammatory cells that infiltrated anterior stroma, recalling phenomena described in conjunctiva in LPS-induced conjunctivitis by Liang et al. [6]. LPS may thus stimulate the blood vessel margin in conjunctiva or in peripheral cornea for inflammatory cell recruitment depending on the injection site (subconjunctival or subcutaneous). In the current model, systemic injection of LPS might induce inflammatory cell diapedesis in vessels derived from the major arterial circle of iris via two concomitant pathways, through uvea to the anterior chamber and through the limbus to the anterior stroma. It is interesting to note that the posterior stroma was completely protected from infiltration between the anterior stroma and the endothelium, suggesting an active defense mechanism inside the cornea, which needs to be described in greater detail.

A better understanding of the HRT II images, thanks to these two new tools, will be useful to develop this noninvasive technique in clinical practice and to investigate endothelial alterations in uveitis. The routine in vivo CM examination of keratic precipitates in patients with uveitis could provide information on the etiology and the degree of inflammatory activity as Wertheim et al. suggested [4]. Furthermore, the advancements in the knowledge of in vivo CM will help to develop in vivo animal experiments in the future to reduce the number of experimental animals used in accordance with the current guidelines.
